# Full-Scale Experimental and Field Investigations into Expansion Mechanism of Foamed Polyurethane and its Lifting Behaviors for Repair and Maintenance of Railway Slab Track Systems

**DOI:** 10.3390/polym16030404

**Published:** 2024-01-31

**Authors:** Zhichao Huang, Qian Su, Ting Liu, Junjie Huang, Xun Wang, Sakdirat Kaewunruen

**Affiliations:** 1 School of Civil Engineering, Southwest Jiaotong University, Chengdu 610031, China or zxh217@student.bham.ac.uk (Z.H.); tmsq@home.swjtu.edu.cn (Q.S.); junjiehuang@swjtu.edu.cn (J.H.); wangxun76@swjtu.edu.cn (X.W.); 2 Department of Civil Engineering, School of Engineering, University of Birmingham, Birmingham B15 2TT, UK; 3 China Railway Construction Kunlun Investment Group Co., Ltd., Chengdu 610040, China; liuting1@crcckl.com

**Keywords:** high-speed railway, slab track, foamed polyurethane, expansion mechanism, lifting behaviors, laboratory tests, settlement repair

## Abstract

Excessive settlement of the subgrade seriously reduces the service quality of slab tracks and threatens trains’ running safety. While the utilization of foamed polyurethane is recognized as an effective solution, previous research on its expansion mechanism and its impact on track lifting requires further refinement. Accordingly, a series of full-scale tests, including expansion force tests on foamed polyurethane with diverse qualities and lifting tests of polyurethane grouting with varied qualities on the track structure, have been conducted. The expansion development process of foamed polyurethane is meticulously elucidated, and key expansion parameters are analyzed. Simultaneously, this research explores the lifting behavior of foamed polyurethane grouting under the slab tracks, yielding new insights into essential lifting parameters for track formation repair and maintenance. Based on the experimental data, this study proposes new empirical formulas to comprehensively describe both the expansion mechanism of foam polyurethane and its lifting behavior under the slab tracks. The outcomes of this research offer a new breakthrough for the design of lifting mechanism for maintaining slab track structures through the utilization of foam polyurethane slurry grouting, such as determining the optimal grouting quantity. In addition, these results are instrumental to the evaluation of lifting effects and service life, enhancing the circular economy of railway track systems.

## 1. Introduction

**Figure 1:**
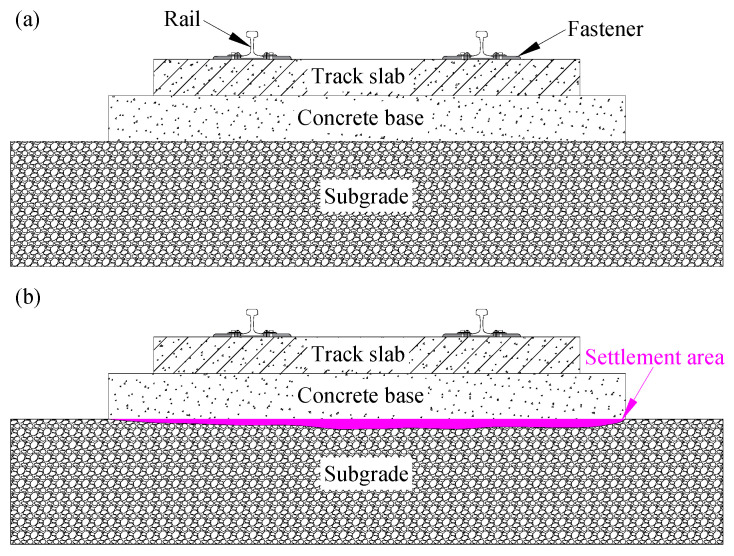
The slab track system; (**a**) normal state; (**b**) settlement.

As of the end of 2022, China’s high-speed railways span a total length of 42,000 km, as reported by the National Railway Administration of the People’s Republic of China. Nearly 70% of these railways are designed for the slab track, due to its notable stability and robust integrity. Illustrated in Figure 1a, the typical slab track system in high-speed railways (HSR) comprises the upper slab track plate, concrete base, and subgrade infrastructure, in which the surface layer and the bottom layer of the subgrade bed are filled with grade-broken stones and group A or B materials, respectively.

The stiffness of the track structure is much greater than that of the subgrade. This means that once the settlement occurs in the foundation, due to the stiffness difference, the track structure fails to undergo coordinated deformation with the subgrade. Consequently, as shown in Figure 1b, this weakens the contact between the track and subgrade, potentially leading to a loss of subgrade support, jeopardizing running safety, and causing damage to the track system. For instance, a simulating analysis [1] of the CRTS II slab track reveals that even a five millimeter subgrade settlement (with a settlement wavelength of 10 m) results in damage and a gradual loss of longitudinal tensile bearing capacity of the slab structure. Differential subgrade settlements contribute to track irregularities and deterioration [2,3,4,5], thereby adversely impacting the dynamic response of the track system during train operations. Various studies [6,7,8,9] suggest that as settlement amplitudes increase, dynamic wheel–rail interactions, car vibrations, and track structure vibrations intensify, posing threats to the comfort and safety of train operations.

**Figure 2:**
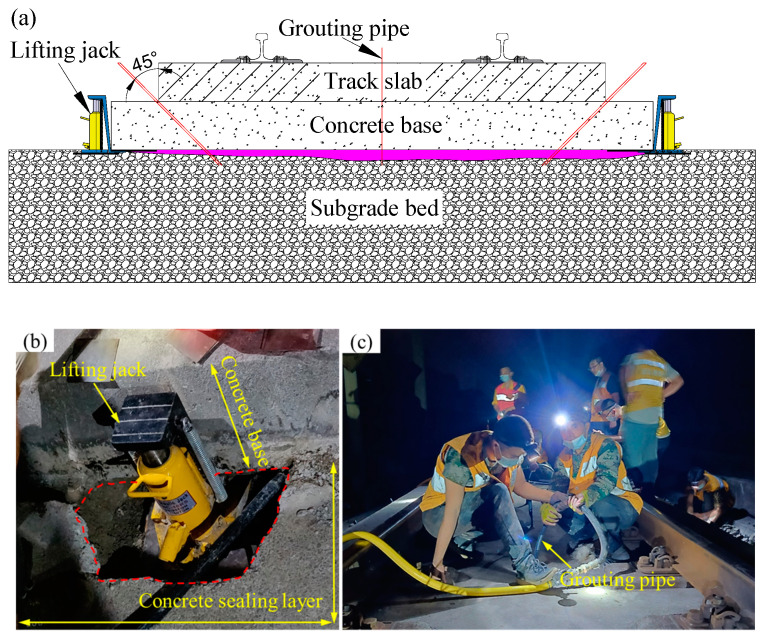
Lifting the slab track by using lifting jacks; (**a**) illustration of the method, (**b**) placing the lifting jack (Weituo-Jingkou Railway), and (**c**) grouting into the bottom of the concrete base (Weituo-Jingkou Railway).

**Figure 3:**
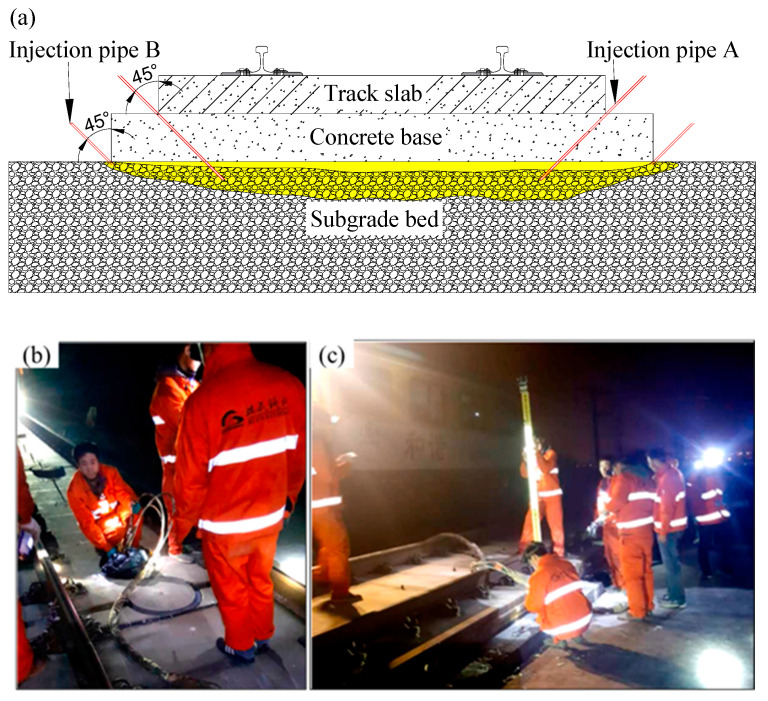
Lifting the slab track by grouting foam polyurethane; (**a**) illustration of the method, (**b**) injecting polyurethane, and (**c**) leveling monitoring of the rail surface.

To ensure the smoothness and stability of slab tracks, China’s HSR design code [10] stipulates that post-construction settlement of the subgrade should not exceed 15 mm. Despite this, during the operation period of the HSR, factors such as groundwater [11,12] and mud pumping [13,14] still can induce uneven subgrade settlement, causing the strong vibration of the track structure under the train load, particularly in coastal and soft soil areas. To maintain the service amount, China’s HSR maintenance criteria [15] mandates that for lines with speeds exceeding 250 km/h, if the settlement wavelength ranges from 1.5 m to 42 m and the vertical settlement exceeds 8 mm in a specific area, repairs are required. Traditional methods involve adding height adjustment pads to the fastener system or lifting the track using jacks. However, it should be noted that the maximum adjustment height of the first method is no more than 26 mm according to the China’s HSR maintenance criteria [15]. As shown in Figure 2, the author’s research team successfully employed the latter traditional method to repair settlement lines in the Weituo-Jingkou Railway. However, this method can damage the integrity of the track system and the conduction process is complex. By contrast, as shown in Figure 3, lifting the slab track by grouting foamed polyurethane can minimize disturbance to the track structure and simplifies the repair process. The essence of this technology is that polyurethane slurry is grouted into a depth ranging from 0.10 m to 0.05 m of the subgrade bed, and then a polyurethane slurry undergoes a combination reactions and expansions, thus the slab structure is lifted. This means that the expansion mechanism of foamed polyurethane plays a crucial role in the effectiveness of this settlement repair method.

Mixing components such as isocyanates, polyols, catalysts, chain extenders, and crosslinkers in specific mass ratios enables the rapid synthesis of polyurethane. This material has excellent properties [16], including high porosity, is lightweight, and has a superior anti-deformation capacity. Many research results [17,18,19,20,21,22,23,24] find that this material has perfect mechanical properties, and laboratory tests and numerical analyses reveal the material’s effectiveness in improving the mechanical properties of sand [25], expansive soil [26], loess soil [27], and gravels [28]. Numerous studies [14,29,30,31,32,33,34,35] demonstrate that by adjusting the mix ratio and density, polyurethane proves beneficial in maintaining the performance of railway foundations. Applications include treating mud pumping, strengthening sleepers and subgrade beds, and solidifying ballasted tracks. These findings indicate that controlling the mix ratio and density ensures that polyurethane meets the compressive strength, tensile strength, and dynamic strength requirements outlined in the HSR design code. This implies that polyurethane layers or mixed gravel layers can effectively support track structures. Additionally, a full-scale model [36] and in-site tests [37] have proven the effectiveness of lifting the track structure by grouting a foamed polyurethane slurry. Moreover, long-term dynamic loading tests have shown that the dynamic response of the slab track has been improved after grouting. However, the concealment and randomness of polyurethane grouting in the interlayer between the concrete base and subgrade bed present challenges to the application of this technology. Moreover, the relationship between the grouting parameters, including grouting quantity, grouting duration, and lifting displacement, remains unclear. Polyurethane grouting for slab track lifting presently relies on experience and real-time observation.

In this paper, a series of expanding force tests is constructed to explore the expansion mechanism with different quantities of the foamed polyurethane. Subsequently, a full-scale slab track model is established, and foamed polyurethane grouting is performed. Based on testing data, the lifting mechanism of polyurethane with different grouting quantities on the slab track is discussed. Empirical formulas are proposed to describe the expansion process of the foamed polyurethane and lifting process by foamed polyurethane grouting on the slab track. The research findings presented in this paper serve as valuable references for the design and assessment of lifting slab tracks employing polyurethane grouting techniques. The insights will improve lifecycle asset management and circular economy practices of high-speed railway track systems.

## 2. Expansion Properties of Polyurethane

### 2.1. Preparation of Polyurethane and Testing Procedure

The foamed polyurethane utilized for lifting the slab track was synthesized with two component slurries in a certain proportion. Generally, component A was isocyanate, while component B comprised a mixture of polyether polyol and various additives, including a catalyst, chain extension crosslinking agent, foaming agent, and foam stabilizer. In this study, the types and physical properties of selected isocyanates and polyether polyol are shown in Table 1a. The catalyst, chain extension crosslinking agent, and foam stabilizers employed were triethylenediamine (TEDA), butane-1,4-diol (BDO), and polyether-modified polysiloxane (PMP), respectively. The physical properties of these different additives provided by the manufacturers are summarized in Table 1b,c. Notably, water was chosen as the foaming agent. The synthesis ratio of foamed polyurethane was *m*_PAPI_:*m*_MDI4110_:*m*_POP2140_:*m*_TEDA_:*m*_BDO_:*m*_PMP_:*m*_w_ = 50:50:200:22.5:2.5:10:4, as set in this research.

**Figure 4:**
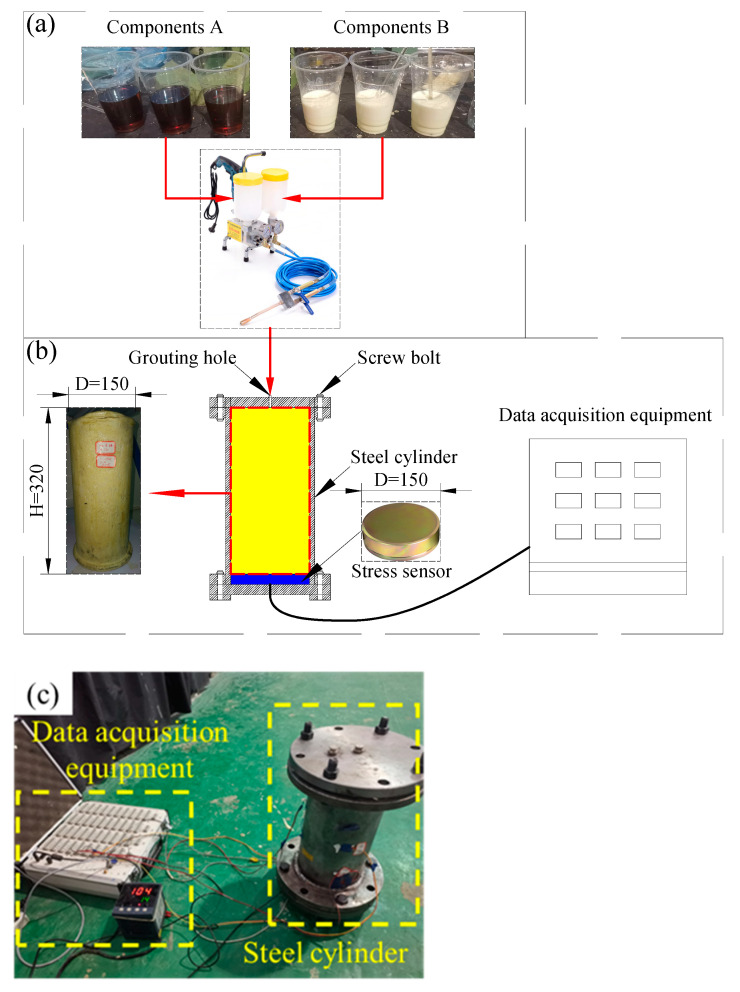
Expansion force testing of foamed polyurethane; (**a**) preparation of foamed polyurethane, (**b**) testing equipment (mm), and (**c**) a physical picture.

The foamed polyurethane preparation process is depicted in Figure 4a. Firstly, component A and component B slurries were prepared according to the designated mix ratio and injected into separate barrels, respectively. Subsequently, using the grouting pump, component A and component B were uniformly mixed and rapidly grouted into the steel cylinder. Once both slurry types were completely injected into the steel cylinder, the grouting hole was sealed, and data of grouting durations and expansion force collections commenced. Notably, the steel cylinder, with an inner diameter of 15 cm, a net height of 32 cm, and a wall thickness of 2 cm, had its top and bottom sealed by flanges, setting the stress sensor on the top surface of the bottom flange. The test equipment is shown in Figure 4a,b. Ten different densities were designed: 0.110 g/cm^3^, 0.140 g/cm^3^, 0.190 g/cm^3^, 0.210 g/cm^3^, 0.250 g/cm^3^, 0.300 g/cm^3^, 0.350 g/cm^3^, 0.400 g/cm^3^, 0.450 g/cm^3^, and 0.530 g/cm^3^, corresponding to grouting foamed polyurethane quantities of 622.0 g, 791.7 g, 1074.4 g, 1187.5 g, 1413.7 g, 1696.5 g, 1979.2 g, 2261.9 g, 2544.7 g, and 2997.1 g, respectively.

### 2.2. Testing Results

**Figure 5:**
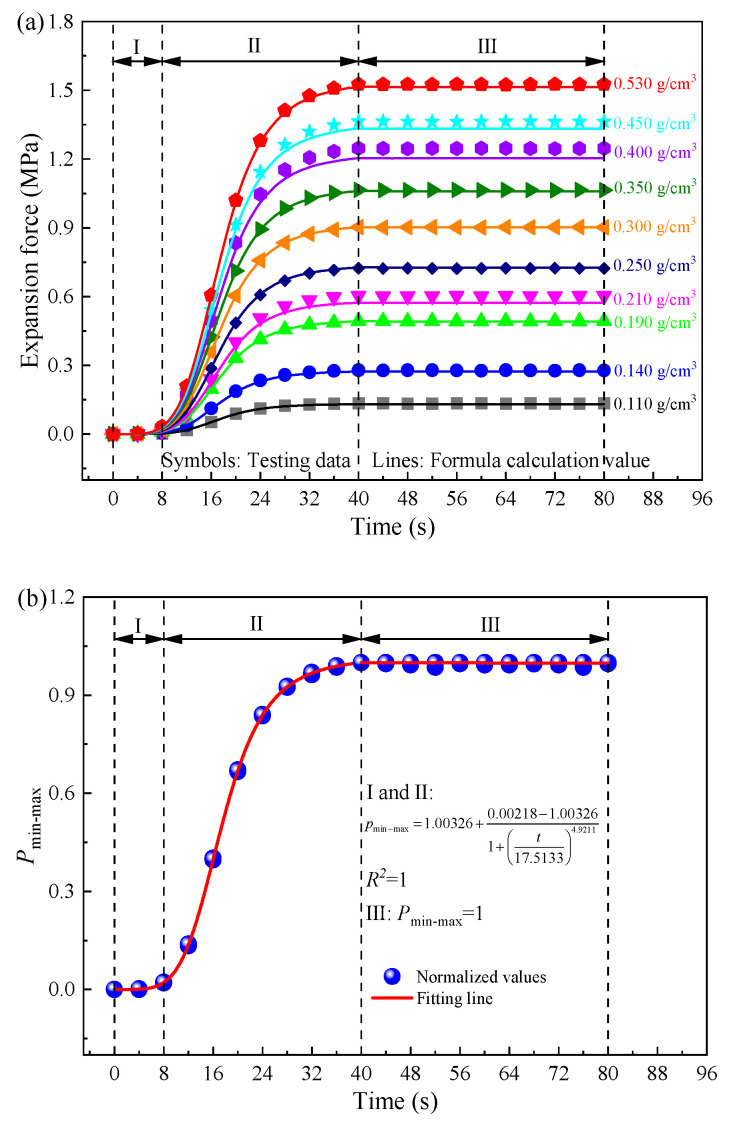

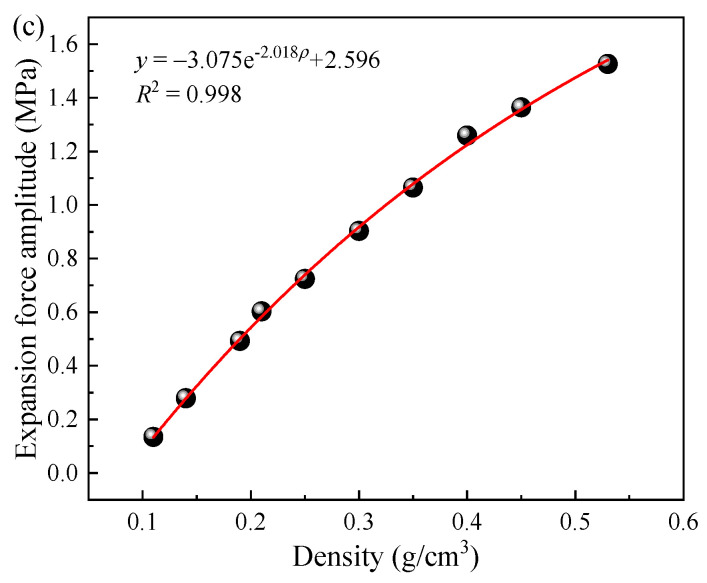
Expansion performances of the polyurethane; (**a**) expansion pressure–time history, (**b**) Min-Max normalization, and (**c**) relationship between the expansion pressure amplitudes and the density of the polyurethane.

Expansion time history curves of foamed polyurethane with different densities are depicted in Figure 5a. Remarkably, although the final expansion pressure value increased with density, the expansion development process of foamed polyurethane remained consistent. Thus, the Min-Max normalization method was used to eliminate the influence of numerical values and discuss the expansion mechanism of foamed polyurethane. Specifically, the normalization values can be calculated using the following equation:

(1) $$p^{\prime }=\frac{p_{i}-p_{\min}}{p_{\max}-p_{\min}},$$

where *p*′ represents the normalized value of the foamed polyurethane expansion force, *p*_i_ is the testing value of the foamed polyurethane expansion pressure at any time for a specific density, and *p*_max_ and *p*_min_ denote the maximum and the minimum values of the expansion pressures of foamed polyurethane for that density. The normalized results of foamed polyurethane expansion pressures are plotted in Figure 5b.

Before delving into the expansion mechanism of foamed polyurethane, it is essential to understand the chemical foaming reaction principle underlying foamed polyurethane. In this study, foamed polyurethane was synthesized using the One-Step Method, wherein the polycondensation reaction between isocyanates and polyols, alongside the bubble production reaction between isocyanates and water, occurs concurrently. Facilitated by catalysts and chain extenders, the polycondensation reaction forms polyurethane chains [38,39]. Simultaneously, with the aid of bubble stabilizers, isocyanates react with water, generating CO_2_ [38,39], which is uniformly dispersed among the polyurethane chains. Upon completion of the reaction, polyurethane with porous characteristics is formed. Notably, the process of bubble formation corresponds to the development of the expansion pressure. The typical chemical reaction formula for this method is as follows:

(2) $$R-NCO+HO-R^{\prime }+H_{2}O=R-NH-COO-R^{\prime }+CO_{2}↑,$$

Continuing the analysis of the foamed polyurethane expansion mechanism based on Figure 5a,b, it is evident that the expansion process unfolds in three distinct stages. In stage I, expansion pressure is not generated until 8 s, which means the bubble production reaction does not occur until the time comes to 8 s. Consequently, stage I is deemed the initial reaction stage, with the initial reaction time being 8 s. Then, it transitions into stage II, characterized by the rapid growth of the expansion pressure. Specifically, from 8 s to 32 s, the expansion pressure rapidly increases, while the increase rate slows down after 32 s. When the time comes to 40 s, the expansion pressure reaches the maximum value, which is also the final expansion pressure. This also indicates that the bubble production reaction mainly occurs during the 8–40 s time period. Furthermore, in this stage, the growth rate of the expansion pressure exhibits an escalating trend with increasing density (or grouting quantity). Specifically, within the density range of 0.110 g/cm^3^ to 0.250 g/cm^3^, growth rates range from 0.0041 MPa/s to 0.0222 MPa/s. For foamed polyurethane with densities ranging from 0.030 g/cm^3^ to 0.350 g/cm^3^ and 0.400 g/cm^3^ to 0.530 g/cm^3^, the corresponding increase rates range from 0.0276 MPa/s to 0.0326 MPa/s and 0.0381 MPa/s to 0.0467 MPa/s, respectively. In addition, the higher the density of foamed polyurethane, which means a greater grouting quantity, the greater its final expansion pressure. Finally, after 40 s, it is in stage III, the solidifying stage. In this stage, expansion pressures fluctuate around the maximum values of the expansion pressure (also the final expansion pressure) before stabilizing. This suggests that foamed polyurethane does not undergo shrinkage during the solidification stage, enhancing its stability for use in the subgrade bed supporting the slab track. Remarkably, the expansion duration (*t_r_*) is consistently 40 s, regardless of the foamed polyurethane slurry quantity.

Expanding on the prior analysis, it is discerned that stages I and II collectively represent the entire expansion process of foamed polyurethane, resembling the curve of the Logistic function. Stage III is identified as the stable stage. Mathematical formulations can be postulated to articulate this developmental pattern, as delineated below:

(3) $$f(t)=p_{f}+\frac{p_{s}-p_{f}}{1+{\left(\frac{t}{t_{0}}\right)}^{\mu_{1}}}\;(0\leq t\leq t_{f}),$$

(4) $$f(t)=p_{f}\;(t>t_{f}),$$

where *p_f_* and *p_s_* (MPa) signify the final values and initial values of the expansion pressures of foamed polyurethane with a certain density, respectively. In this study, *p_s_* is 0. The coefficient *t* denotes time (s), *t_f_* represents the time point at which expansion ends, while *t*_0_ and *μ*_1_ represent the inflection point of the curve and slope at the inflection point, respectively. This means that *t*_0_ is the critical time point at which the bubble production reaction transitions from intense to slow, and *μ*_1_ is associated with the bubble production reaction rate at this juncture. Actually, the bubble production reaction is linked to the raw material ratio of the synthesized foamed polyurethane, independent of the quantity of foam polyurethane slurry. Moreover, all data in Figure 5a are normalized by the Min-Max normalization method and plotted in Figure 5b, where it can be found that the expansion process exhibits similarity regardless of the foamed polyurethane slurry quantity. Therefore, through fitting from Figure 5b, the values of *t*_0_ and *μ*_1_ are determined to be 17.5133 and 4.9211, respectively.

As mentioned previously, the expansion pressure exhibits an increase with the rise in the density of cured foamed polyurethane. Drawing from testing data, Figure 5c is formulated to explore this relationship. Through calculation, this can be articulated using a natural exponential function, expressed as:

(5) $$p_{f}=ae^{b\rho }+c,$$

where *p_f_* signifies the final expansion pressure values (MPa), *ρ* represents the density of foamed polyurethane (g/cm^3^), and *a*, *b*, and *c* all denote correlation coefficients. Following fitting with the testing data, the coefficients are determined as −3.075, −2.018, and 2.596, respectively. Furthermore, considering that *ρ* is the mass-to-volume ratio, with a known volume, the final expansion pressure can be calculated based on the grouting quantity using the following equation:

(6) $$p_{f}=ae^{b\left(\frac{m}{V}\right)}+c,$$

where *m* represents the quantity of polyurethane slurry (g) and *V* is the volume (cm^3^). Thus, combining Equations (3)–(6), the empirical mathematical expression of the expansion mechanism of foamed polyurethane is proposed as follows:

(7) $$f(t)=\frac{(-3.075e^{-2.018\left(\frac{m}{V}\right)}+2.596)t^{4.9211}}{17.5133^{4.9211}+t^{4.9211}}\;(0\leq t\leq t_{r}),$$

(8) $$f(t)=-3.075e^{-2.018\left(\frac{m}{V}\right)}+2.596\;(t>t_{r}),$$

where *t* represents any time point during the experimental process (s), *ρ* represents the density of foamed polyurethane (g/cm^3^), and *f*(*t*) denotes the expansion pressure at any time (Mpa). In this study, *t_r_* is 40 s. Therefore, the calculation results of expansion pressures of foamed polyurethane of different quantities can be obtained and plotted in Figure 5a. Obviously, the calculated values closely align with the testing data, with related errors consistently below 7.10%. It is emphasized that while errors in the initial stage might appear slightly larger, given the minute values of expansion pressures, these discrepancies are negligible. Thus, the expansion process of polyurethane foam in a completely confined space can be described using Equations (7) and (8).

## 3. Lifting Behaviors by Polyurethane Grouting to the Slab Track

### 3.1. Model Construction and Testing Procedure

**Figure 6:**
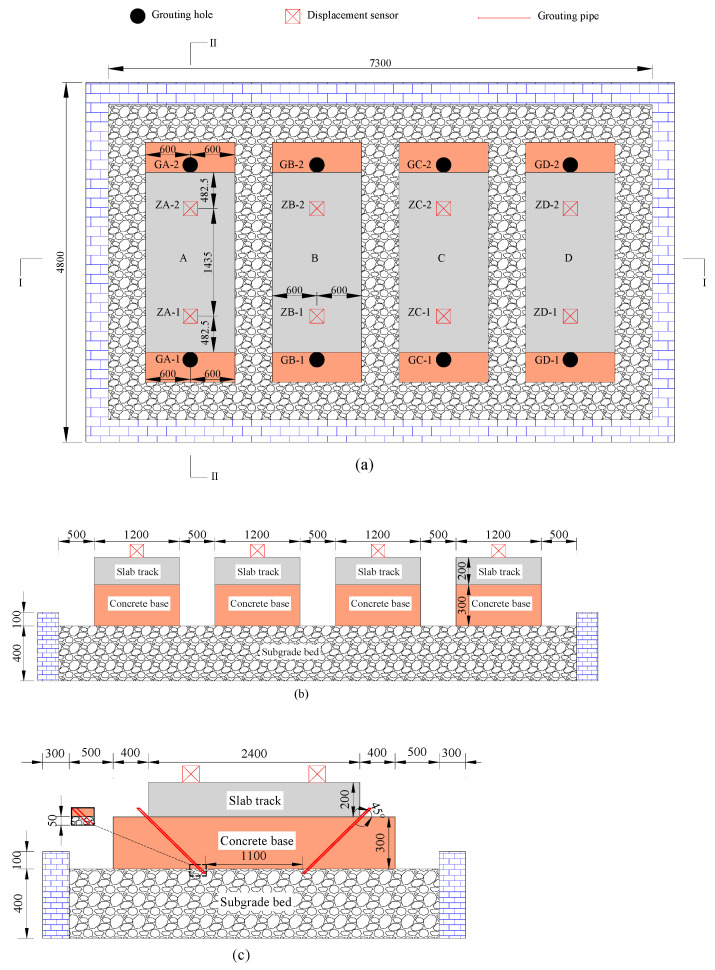
Model of the slab track; (**a**) top view of the layout of the displacement sensors and grouting holes, (**b**) I-I section, and (**c**) II-II section (unit: mm).

**Figure 7:**
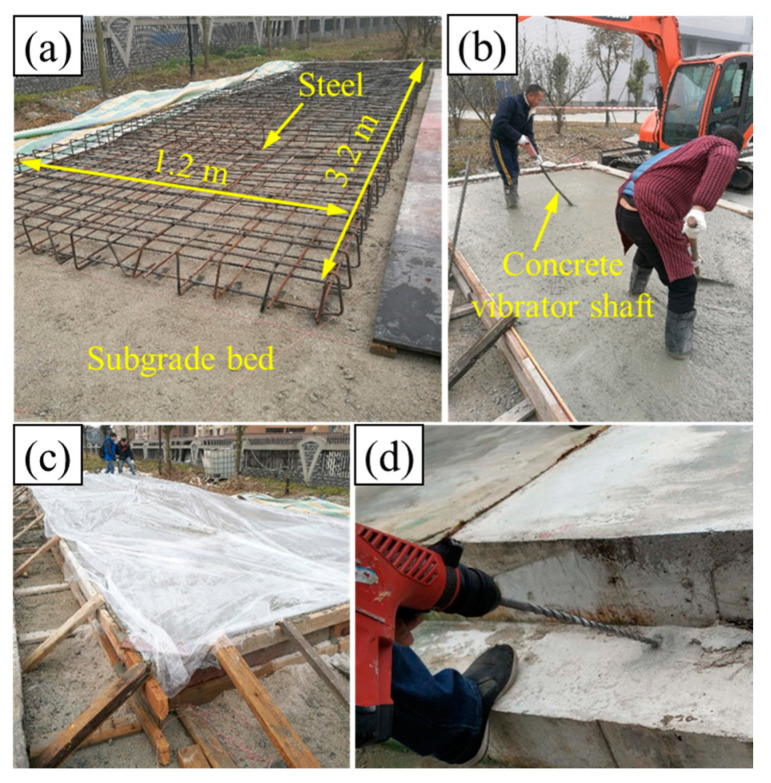
Model construction: (**a**) subgrade bed and the reinforcement cage in E area, (**b**) paving and vibrating of concrete, (**c**) curing by being covered with PE film, and (**d**) drilling the hole.

The full-scale slab track grouting model was constructed in a brick wall box measuring 7.3 m in length, 4.2 m in width, and 0.5 m in height, as illustrated in Figure 6. This model comprised slab tracks, concrete bases, and a subgrade bed filled with well-graded gravel, all constructed following the Chinese high-speed railway design code [10]. It is noteworthy that the slab track was built with concrete (C50) and threaded steel bars with a diameter of 20 mm, and the concrete base plate was built with concrete (C15). In this study, four track structures with a length of 1.2 m were constructed for lifting tests of different grouting quantities. The construction process is detailed in Figure 7. The compaction degree of the subgrade bed was set to 0.97.

The track structure was cast in place directly on the surface of the subgrade bed, as illustrated in Figure 7a. Following concrete paving and vibration (as shown in Figure 7b,c), the surface of the slab track was covered with PE film for a curing period of 7 days. Subsequently, grouting holes with a diameter of 25 mm were drilled in each slab track structure, as shown in Figure 7d. Grouting pipes with a diameter of 25 mm were then inserted into all the holes, with the end of the grouting pipe positioned approximately 5 cm below the surface of the subgrade bed, as illustrated in Figure 6c. Displacement sensors were installed on the surface of each slab track structure, and the distribution of the grouting holes and displacement sensors is plotted in Figure 6a–c.

**Figure 8:**
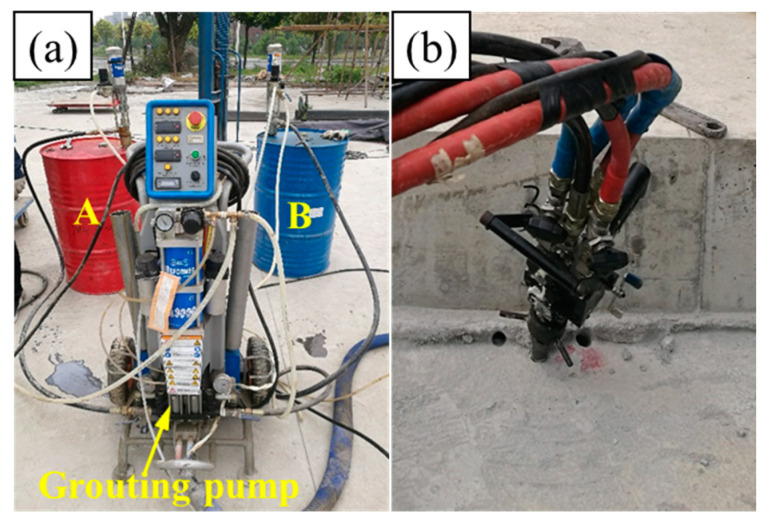
Grouting foamed polyurethane; (**a**) grouting equipment, and (**b**) grouting.

In this study, the total quantity of foamed polyurethane grouted into the slab track structures A, B, C, and D were 3.0 kg, 4.5 kg, 6.0 kg, and 7.5 kg, respectively. For structures A, B, C, and D, the grouting quantity in a single hole was 1.50 kg, 2.25 kg, 3.00 kg, and 3.75 kg, respectively. Synchronous grouting on both sides of the slab track was conducted to ensure balance during the lifting process. Specifically, as shown in Figure 8, the components A and B were first prepared in sufficient quantities following the synthesis ratio mentioned earlier. Subsequently, these components were simultaneously grouted into the subgrade bed through the holes on both sides of the slab track using a grouting pump. The grouting pressure was set to 5 MPa, and once the set grouting quantity was reached, the grouting was stopped. Data collection commenced from the beginning of the grouting process, with a data collection interval set to 1 s. When the displacement tended to stabilize, data collection was stopped.

### 3.2. Testing Results and Discussion

**Figure 9:**
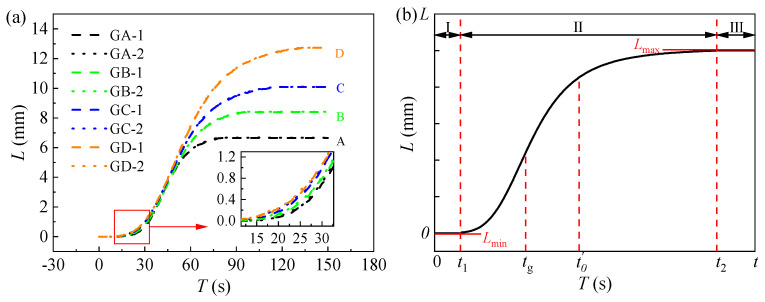
Time history curve of lifting displacement; (**a**) different testing positions, and (**b**) lifting mechanism.

The variation in the laws of lifting displacement with time history for each testing point are plotted in Figure 9. It is obvious that the related errors of the test values for lifting displacement at different measuring points of the same structure are very small. Specifically, the related errors between the testing values at GA-1 and GA-2, at GB-1 and GB-2, GC-1 and GC-2, and GD-1 and GD-2 are all less than 2.00%. This observation illustrates the efficacy of symmetrical synchronous grouting on both sides in ensuring uniform lifting of the slab track structure. Furthermore, it can be found that the increase laws with time are similar, although the lifting displacement amplitudes (the final lifting displacement values) are different. This phenomenon is illustrated by Figure 9b.

The lifting mechanism of the grouting foamed polyurethane can be divided into three stages. Stage I ranges from the grouting start to *t*_1_ and is the diffusion and initialization reaction stage. In this stage, the foamed polyurethane slurry gradually diffuses in the subgrade bed under the influence of grouting pressure. Once reaching the critical point of the combination reaction, the slurry gradually solidifies and expands. As mentioned before, the expansion pressure increases with time; thus, lifting only occurs when the expansion pressure reaches the self-weight of the track structure, i.e., at time *t*_1_. During stage I, the lifting displacement is the smallest (*L*_min_), with a value of 0. It then enters stage II, the lifting stage, where the grouting continues, and the slurry undergoes combination reactions and foaming, resulting in a rapid increase in the expansion pressure and lifting of the slab track until the grouting stops at the moment of *t_g_*. After that, the slurry gradually stops diffusing but continues to undergo a combination reaction, thereby continuing to rapidly lift the slab track. Subsequently, in the time period *t*′_0_ to *t*_2_, the combination reaction and foaming of the slurry gradually slow down, the foamed polyurethane slurry solidifies gradually, and the lifting displacement increases slightly. At *t*_2_, the combination reaction and foaming of the foamed polyurethane are completed, lifting stops, and the lifting displacements reach the maximum values (*L*_max_), which are also the final lifting displacement values (*L_f_*). *t*_2_ marks the end time of lifting. Finally, stage III is entered, where the lifting displacement fluctuates around the values of *L_f_* in the initial period and then tends to stabilize at *L_f_* gradually. The track structure is supported by the cured polyurethane and the subgrade bed.

**Figure 10:**
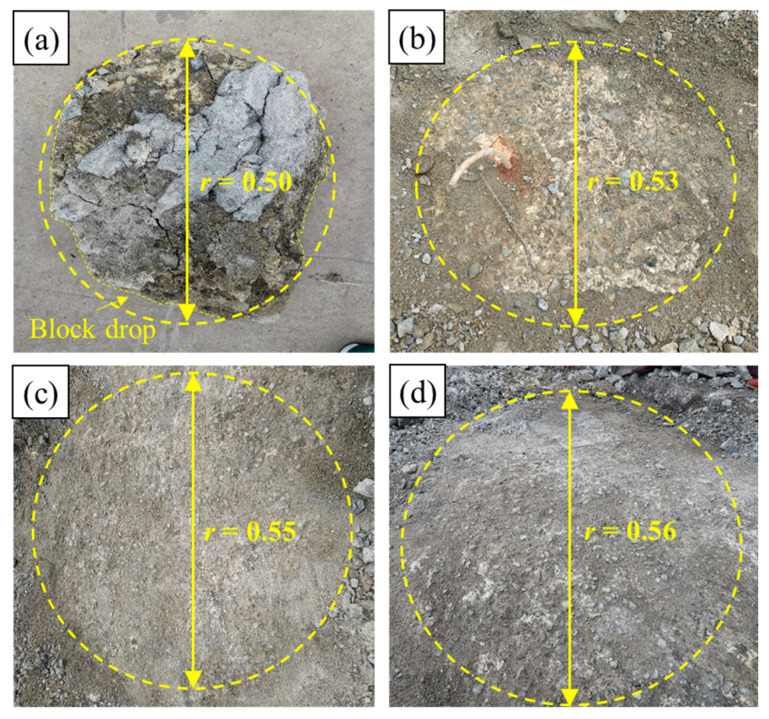
Diffusion radius: (**a**) 1.50 kg, (**b**) 2.25 kg, (**c**) 3.00 kg, and (**d**) 3.75 kg (unit: m).

After 2 h of completing the lifting, the slab track structure was removed, and the diffusion radius under different grouting quantities can be observed, as shown in Figure 10. Notably, the diffusion area of foamed polyurethane grouting in the well-graded gravel resembles a circular shape, although some solid blocks within the diffusion area have detached. The key values from the grouting tests, including total grouting quantity (*m_t_*), grouting duration (*t_g_*), final lifting displacements (*L_f_*), diffusion radius (*r*), initial lifting time (*t*_1_), and lifting duration (*t*_2_), were obtained and are presented in Table 2. The data illustrates that, under consistent grouting pressure and medium, the final lifting displacements are positively correlated with the total grouting quantity. Moreover, due to the time-dependent viscosity of the foamed polyurethane slurry, and the friction resistance of the well-graded gravel, an increase in grouting quantity leads to a prolonged grouting duration, assuming the grouting pressure remains constant. In the same grouting environment, the initiation time for lifting, with different total grouting quantities, is nearly identical, emphasizing that the initial reaction time of foamed polyurethane is primarily linked to its composition. Furthermore, the observed end times for lifting exceed the theoretical values, which account for the sum of the grouting duration and foamed polyurethane synthesis reaction duration (in the steel cylinder). This suggests that the combination reaction of foamed polyurethane slurry in well-graded gravel with a certain compaction degree is considerably more intricate than that in the steel cylinder.

**Figure 11:**
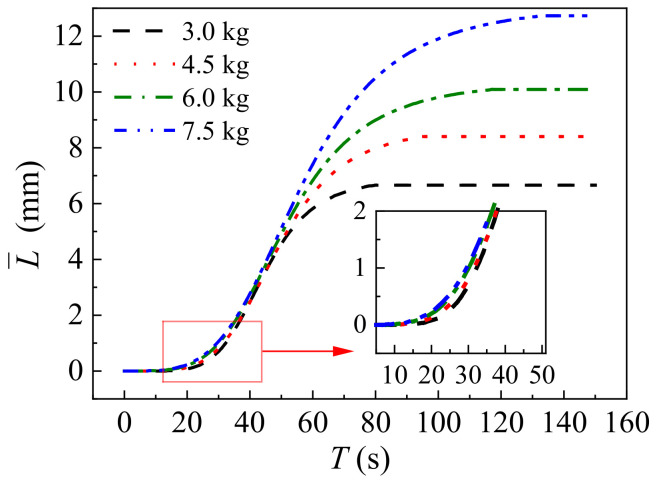
Time history curve of the mean lifting displacement ($\bar{L}$—*T*).

As previously mentioned, the error between the lifting values on both sides (A, B, C, and D) is minimal. Consequently, the average values ($\bar{L}$) can be calculated using the following equation to further discuss the lifting mechanism:

(9) $$\bar{L}=\frac{L_{1}^{i}+L_{2}^{i}}{2},$$

where $L_{1}^{i}$ and $L_{2}^{i}$ (mm) represent the testing values of the lifting displacement on both sides of the track slab at any time. The resulting curve of $\bar{L}$ − *T* is plotted in Figure 11. Additionally, the non-linear relationship between the lifting displacement and time is attributed to the expansion mechanism of foamed polyurethane. It is observed that both stages I and II in all time history curves of lifting displacement resemble the curve of the logistic function, and in stage III, the lifting displacement stabilizes at *L_f_*. Hence, the following mathematical formulas can be given out to describe the lifting behavior of foamed polyurethane grouting:

(10) $$f(t)=L_{\max}+\frac{L_{\min}-L_{\max}}{1+{\left(\frac{t}{t_{0}^{\prime }}\right)}^{\mu_{2}}}\;(0\leq t\leq t_{2}),$$

(11) $$f(t)=L_{\max}\;(t_{2}<t),$$

where *t* represents time (s), and *t*’_0_ and *μ*_2_ denote the inflection point and slope at the inflection point during the growth stage of the curve, respectively. In this study, *L*_min_ is 0, and *L*_max_ is related to the total grouting quantity, as mentioned before. Values of *t’*_0_ and *μ*_2_ can be obtained by fitting the data in Figure 11, and are plotted in Table 3.

**Figure 12:**
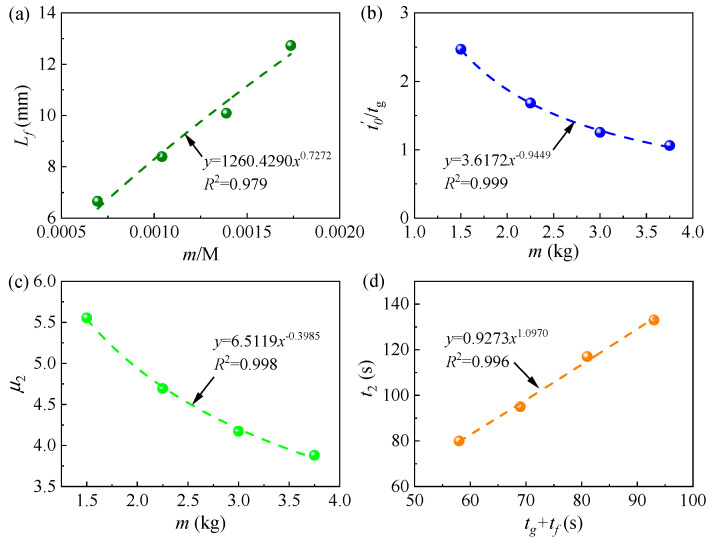
Parameters analyses; (**a**) ***L_f_***, (**b**) *t*′_0_, (c) *μ*_2_, and (d) *t*_2_.

Based on previous analysis, it is apparent that the expansion pressure is closely linked to the quantity of foamed polyurethane, and considering that the self-weight of the track structure plays a critical role in the lifting process, the ratio of grouting quantity to the self-weight of the track structure is introduced to evaluate the final lifting displacement value. Additionally, it is observed that values of *t*′_0_ are greater than those of *t_g_* in all curves. In stage II, during the period from *t*_1_ to *t_g_*, the slurry expands while diffusing, and when the grouting is completed, that is, after *t_g_*, the slurry still diffuses for a short period, while the diffusion of the slurry affects the foaming of the slurry. As mentioned previously, *t*′_0_ is the inflection point. Based on these analyses, it can be proposed that *t*′_0_ correlates with *m* and *t_g_*. It is evident that the duration of lifting is closely associated with both the grouting and expansion durations of foamed polyurethane. Building on the aforementioned analyses, the following equations are proposed to discuss the parameters in Equation (5):

(12) $$L_{f}=a_{1}{\left(\frac{m_{t}}{M}\right)}^{b_{1}},$$

(13) $$\frac{t_{0}^{\prime }}{t_{g}}=a_{2}m^{b_{2}},$$

(14) $$\mu_{2}=a_{3}m^{b_{3}},$$

(15) $$t_{2}=a_{4}{\left(t_{g}+t_{r}\right)}^{b_{4}},$$

where *m* and *M* (kg) signify the foamed polyurethane grouting quantity in a single hole and the self-weight of the slab track, respectively. *t_r_* denotes the duration of foamed polyurethane expansion, while *a_i_* and *b_i_* represent correlation coefficients. In this study, *M* is 4320 kg and *t_r_* is 40 s. Therefore, the utilization of Equations (12)–(15) allows the generation of Figure 12, illustrating the values of all coefficients, as depicted in the figure. Specifically, in this investigation, *a*_1_ and *b*_1_ are 1259.96 and 0.7271, respectively. Similarly, *a*_2_ and *b*_2_ are found to be 3.6172 and −0.9449, respectively, while *a*_3_ and *b*_3_ are 6.5119 and −0.3985, and *a*_4_ and *b*_4_ are 0.9273 and 1.0970, respectively. All R-Square values exceed 0.95, indicating the reliability of the coefficients obtained through fitting testing data.

**Figure 13:**
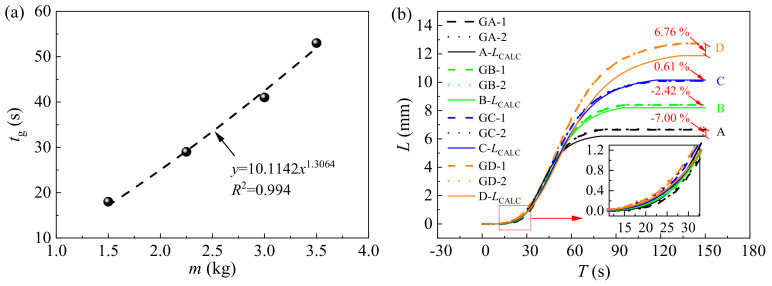
Lifting mechanism analyses; (**a**) analyses of parameter *t_g_*, and (**b**) calculated values and testing values of the lifting displacement.

In the grouting process, the monitoring values of *t_g_* are crucial for evaluating the lifting effect. When the grouting environment remains unchanged, the grouting duration can be estimated based on the grouting quantity. In this study, through calculations, as depicted in Figure 13a, it was observed that the relationship between *t_g_* and *m* can be expressed by the following equation:

(16) $$t_{g}=a_{5}m^{b_{5}},$$

where *a*_5_ and *b*_5_ are coefficients, determined to be 10.1142 and 1.3064, respectively, in this study. Utilizing Equation (16), the calculated values of *t_g_* for grouting quantities of 1.50 kg, 2.25 kg, 3.00 kg, and 3.75 kg are 17 s, 29 s, 42 s, and 57 s, respectively. The related errors between the calculated and testing values of *t_g_* are −5.56%, 0, 2.44%, and 7.55%, respectively, confirming the reliability of Equation (16). Consequently, values of *L_f_*, *t*′_0_, *μ*_2_, and *t*_2_ can be determined by combining Equation (12) through Equation (16). The calculated values (CALC) and testing values of these parameters are presented in Table 3. The absolute related errors between the test values and calculated values for all parameters range from 0 to 5.66%, demonstrating the reliability of the calculated values. Employing Equations (4)–(16), the time history curve of the lifting displacement with different grouting quantities can be plotted in Figure 13b. It is evident that for structures A, B, C, and D, the maximum related errors between the calculated values and testing values of the lifting displacement are −7.00%, −2.42%, 0.61%, and 6.76%, respectively. This indicates an acceptable level of accuracy; therefore, Equations (5)–(16) are recommended for estimating the quantity of foamed polyurethane grouting required to lift the track slab and for evaluating the real-time lifting development process.

## 4. Conclusions

This paper conducts a thorough examination of the expansion force characteristics of foamed polyurethane and explores the lifting behavior of the foamed polyurethane on slab tracks through grouting lift tests. The following conclusions can be drawn.

(1) The initial reaction time and expansion duration of the foamed polyurethane slurry remain at 8 s and 40 s, respectively. The final expansion pressure and growth rate are positively correlated with the grouting quantity, and an empirical formula is provided to calculate the final expansion pressure based on the grouting quantity.

(2) The expansion process of the polyurethane slurry is categorized into three stages: the initial reaction stage, expansion development stage, and the stable stage. The initial reaction and expansion development stages are described using logical functions, while the stable stage is represented as a constant function. The boundary time point between the expansion development stage and the constant stage is determined as T = 40 s.

(3) The diffusion morphology of the foamed polyurethane slurry in the shallow layer of the subgrade bed surface appears circular. Under constant grouting pressure, the diffusion radius in well-graded gravel with a compaction degree of 0.97 increases non-linearly with the grouting quantity. The starting time of lifting is less affected by the quantity of grouting, while the duration of lifting is related to the grouting quantity and duration. The final lifting displacement value shows an increasing trend with the rise in grouting quantity.

(4) The lifting process exhibits characteristics resembling the approximation curve and can be divided into three stages: the diffusion of the slurry and the initial reaction stage, the continuous lifting stage, and the slurry solidification forming stage, which also is the stable lifting stage. The optimized logical function formula has been proposed to describe the process of uplift development, and a constant function can be used to express the stable stage of uplift. In addition, empirical formulas have been proposed to determine the grouting duration and lifting duration based on the grouting quantity. The insights obtained from this study have already been applied to high-speed railway track maintenance practices in reality. The outcome helps high-speed rail engineers improve lifecycle asset management and circular economy practices in the railway industry.

While this study offers valuable insights into the application of foamed polyurethane for the settlement repair of slab tracks, it acknowledges certain limitations, such as the incomplete explanation of the foamed polyurethane slurry diffusion mode in well-graded gravel, and the influence of spatial constraints conditions on the expansion mechanism of foamed polyurethane. These scientific questions will be addressed in future numerical and theoretical analyses to optimize and refine the inflation mechanism and uplift mode proposed in this paper.

## Figures and Tables

**Table 1:** Physical properties of raw materials.

(a)									
Raw Materials	Hydroxyl Value (mgKOH/g)		Functionality		Viscosity (25 °C) (mPa·s)		Water Content (%)		Acid Value (%)
PAPI	-		2.6~2.7		150~250		-		≤0.03
MDI 4118P	450 ± 20		4~4.5		7000 ± 1000		≤0.15%		-
POP 2140	19.5~22.5		2		7000		≤0.05%		≤0.10
**(b)**									
**Raw Materials**		**Melting Point** **(°C)**		**Boiling Point** **(°C)**		**Flash Point (mPa·s)**		**Purity** **(%)**	
TEDA		158		174		50		99.5	
BDO		20		228		121		99	
**(c)**									
**Raw Materials**	**Specific Weight (25 °C)**		**Surface Tension (25 °C)** **(mN/m)**		**Viscosity (25 °C)** **(mPa·s)**		**Cloud Point** **(°C)**		**Purity** **(%)**
PMP	1.08		23.6		900~1500		45		99.9

**Table 2:** Key values of the lifting testing.

No.	*m_t_* (kg)	*t_g_* (s)	*L_f_* (mm)	*r* (m)	*t*_1_ (s)	*t*_2_ (s)
A	3.0	18	6.66	0.50	19	80
B	4.5	29	8.40	0.53	18	95
C	6.0	41	10.09	0.55	18	117
D	7.5	53	12.73	0.56	16	133

**Table 3:** Values of parameters.

NO.	*L_f_* (mm)			*t*’_0_ (s)			*μ*_2_ (s)			*t*_2_ (s)		
	Test	CALC	RE	Test	CALC	RE	Test	CALC	RE	Test	CALC	RE
A	6.66	6.37	−4.35%	44.4360	41.9211	−5.66%	5.5552	5.5403	−0.27%	80	80	0.00%
B	8.40	8.55	1.79%	48.8082	48.7521	−0.11%	4.6939	4.7137	0.42%	95	96	1.05%
C	10.09	10.54	4.46%	51.4103	53.8009	4.65%	4.1732	4.2032	0.72%	117	115	−1.71%
D	12.73	12.40	−2.59%	56.2549	59.1351	5.12%	3.8809	3.8455	−0.91%	133	134	0.75%

## Data Availability

The data presented in this study are available upon request from the corresponding author or the first author.
